# Transcending risk factors: the implications of redefining diabetes as an immunometabolic disease for infectious disease studies

**DOI:** 10.3389/fimmu.2026.1794628

**Published:** 2026-03-13

**Authors:** Xiujun Zhang, Fan Zhang, Wenjian Li

**Affiliations:** The Affiliated Changzhou Hospital of Xuzhou Medical University, Changzhou Third People’s Hospital, Changzhou, China

**Keywords:** clinical management, diabetes, immune dysregulation, immunometabolism, infectious diseases

## Abstract

This review methodically argues for a theoretical paradigm shift. The text redefines diabetes as an “immunometabolic disorder” and explores its profound implications. Conventional wisdom has long regarded diabetes as a risk factor for infectious disease comorbidity. Nevertheless, this perspective lacks the depth necessary to elucidate the underlying mechanisms involved. Preliminary clinical research suggests that individuals with diabetes have a 2- to 4-fold higher probability of requiring hospitalization due to infections in comparison with individuals who do not have diabetes. During the pandemic, there was a notable surge in severe illness and mortality rates. These findings suggest limitations in the traditional explanatory framework centered solely on glycemic control. The new paradigm focuses on the co-dysregulation of immunity and metabolism, elucidating how factors such as hyperglycemia and lipotoxicity interact to ultimately drive chronic inflammation and immune dysfunction. This framework elucidates the underlying mechanisms that render individuals with diabetes more vulnerable to various pathogens, expedite disease progression following infection, and manifest diminished therapeutic and vaccine responses. The review further posits that this understanding should inform clinical practice. Rather than prioritizing solely glycemic control, a comprehensive approach to management should encompass metabolic regulation, immune assessment, and infection prevention. Public health policies must explicitly categorize diabetic patients as a priority group for protection. Despite the challenges associated with precise subtyping and the development of targeted interventions, the potential benefits of interdisciplinary efforts and precision medicine strategies are noteworthy. A critical imperative for diabetes management is a transition from a blood glucose control-centric approach to a comprehensive intervention strategy aimed at restoring immune-metabolic homeostasis.

## 1 Introduction

The association between diabetes and infection has been recognized in clinical practice for a long time (1, 2). Extensive research indicates that individuals with diabetes are susceptible to multiple infectious diseases, exhibiting significantly higher infection rates, risks of severe disease, and related mortality compared to non-diabetic populations (1–4). For instance, individuals with diabetes have been shown to experience elevated rates of respiratory, urinary tract, skin and soft tissue, and postoperative wound infections (2, 4, 5). Moreover, diabetes has been identified as a significant risk factor for severe disease, increased complications, and poor clinical outcomes in specific infectious diseases, including influenza, severe acute respiratory syndrome coronavirus 2 (SARS-CoV-2), and tuberculosis (6–8). The prevailing explanation for this phenomenon is the direct promotion of pathogen proliferation in a hyperglycemic environment, as well as impaired tissue perfusion, sensory loss, and compromised barrier function resulting from chronic vascular and neurological complications of diabetes. These conditions create a favorable environment for infection (1, 2). However, this framework, which is predicated on clinical manifestations and end-stage complications, has limitations. It fails to explain why patients with similar glycemic control levels exhibit significant individual heterogeneity in infection risk, nor does it elucidate diabetes’s profound impact on immune responses to specific pathogens. The conventional viewpoint views diabetes as a mere “background state” that fosters infection, rather than as a pivotal catalyst that directly contributes to the underlying pathophysiological processes driving infection.

In recent years, the rapid advancement of the interdisciplinary field of immunometabolism has profoundly transformed our understanding of diabetes (9–13). Immunometabolic dysregulation is defined as a state of imbalanced mutual regulation between the immune system and metabolic networks. The manifestation of this condition is characterized by metabolic abnormalities, including hyperglycemia and lipotoxicity, which in turn lead to immune dysfunction. Consequently, immune inflammation disrupts metabolic homeostasis, creating a vicious cycle (9, 11). A mounting body of research suggests that diabetes, particularly type 2 diabetes, extends beyond a mere disorder of glucose metabolism. The condition is characterized by a persistent, low-grade systemic inflammatory state, multifaceted dysregulation of both innate and adaptive immune systems, and direct modulation of immune cells by a metabolic environment shaped by hyperglycemia, advanced glycation end products (AGEs), and lipotoxicity (14, 15). This prompts a reconceptualization of diabetes as an “immunometabolic disease (16).” This definition underscores the inextricable intertwining of abnormalities in the immune system and metabolic disorders, emphasizing their mutual causality in the pathogenesis of diabetes (9, 17, 18).

While the relationship between diabetes and infection is a well-established one, extant research has focused primarily on the explanation of glycemic control and complications, with a paucity of systematic integration of immune-metabolic interactions. This perspective gap hinders our understanding of the root causes of infection heterogeneity in diabetic patients and limits the development of precision intervention strategies. The objective of this review is twofold: first, to integrate recent advances in immunometabolism to propose a unified pathophysiological framework, and second, to elucidate how diabetes, as an immunometabolic disorder, reshapes the infection process. By doing so, it is hoped that new insights will be provided for clinical practice and public policy. Subsequent sections will sequentially address the pathophysiological mechanisms of immunometabolic disorder, its reshaping of specific infectious disease clinical phenotypes, and the resulting implications for clinical management and public health. This integrated framework aspires to furnish a more comprehensive theoretical foundation for both research and clinical practice in this domain.

## 2 Pathophysiological basis of immunometabolic dysregulation

The fundamental principle underlying immunometabolic disorder in diabetes is the presence of an interconnected, self-reinforcing network of pathophysiological alterations (16). At the core of this network are disruptions to metabolite homeostasis and multidimensional dysregulation of immune function, which mutually reinforce each other in a vicious cycle. The observed phenomenon extends beyond mere energy supply abnormalities, suggesting a more profound underlying biological concern: the presence of abnormal metabolic intermediates functions as a persistent endogenous danger signal, thereby modulating the host’s immune response profile (19).

### 2.1 Metabolites as immunoregulatory signals

The abnormal metabolite pool has been identified as the initial driver of immune dysregulation. Persistent hyperglycemia, the hallmark of diabetes, has numerous immunological effects. At the cellular level, the hyperglycemic environment directly impairs immune cell function through multiple pathways, including mitochondrial oxidative stress, increased flux through the hexosamine pathway, and increased protein kinase C activation (20). For instance, neutrophils exhibit reduced chemotaxis, phagocytosis, and neutrophil extracellular traps (NETs) formation—a “hyperglycemia-induced functional paralysis” that diminishes their bacterial clearance efficiency (21). Monocyte/macrophage phenotypic polarization is also disrupted, favoring pro-inflammatory (M1) differentiation while exhibiting defective phagocytosis and antigen presentation—a state of “dysfunctional activation (22, 23).” AGEs have been identified as a significant pathological mediator. The binding of AGEs to cell-surface receptors, such as the receptor for advanced glycation end products (RAGE), results in persistent activation of downstream signaling pathways. These pathways include nuclear factor-κB and mitogen-activated protein kinases, which drive the expression of pro-inflammatory cytokines and adhesion molecules. This process constitutes the molecular foundation of chronic low-grade inflammation, a hallmark feature of diabetes (24). Furthermore, abnormalities in lipid metabolism that accompany insulin resistance—particularly elevated circulating free fatty acid levels—mimic pathogen-associated molecular pattern stimulation by activating Toll-like receptors (especially TLR2 and TLR4) on immune cells like macrophages and dendritic cells, thereby inducing chronic inflammatory responses (23, 25). Of particular significance is the disruption of fatty acid metabolism, which interferes with the energy metabolic reprogramming of immune cells. For instance, it has been demonstrated that free fatty acids impairs macrophages’ ability to transition from oxidative phosphorylation to aerobic glycolysis (26, 27). This transition is critical for the effective activation and execution of macrophage bactericidal functions.

### 2.2 Systemic dysregulation of the immune system

The sustained activation of these metabolic signals triggers a comprehensive and substantial functional transformation of the immune system. At the level of innate immunity, this phenomenon is characterized by a paradoxical state of “inflammatory aging” or “immune paralysis,” marked by a sustained elevation in basal inflammation levels coupled with diminished responsiveness to new infections. The overactivation of NLR family pyrin domain-containing 3 (NLRP3) inflammasome is a fundamental aspect of this phenomenon (28, 29). Endogenous danger signals, including hyperglycemia, AGEs, free fatty acids, and reactive oxygen species, have been shown to activate the NLRP3 inflammasome, which subsequently activates caspase-1. This process subsequently facilitates the maturation and release of pro-inflammatory cytokines interleukin-1 beta (IL-1β) and interleukin-18 (IL-18), thereby triggering pyroptosis (28, 29). A similar disruption is observed in the adaptive immune system (30, 31). CD4(+) T lymphocyte differentiation becomes skewed, potentially impairing the function of T helper type 1 (Th1) and T helper type 17 (Th17) cells and affecting defenses against intracellular pathogens and fungi (32, 33). Concurrently, the immunosuppressive function of regulatory T cells is compromised, leading to inadequate control of inflammation (34). Furthermore, CD8(+) T cell toxicity and the capacity to form memory may also decline (35). B lymphocyte dysfunction is characterized by impaired antibody response quality, including defects in antibody affinity maturation, abnormal class switching, and reduced vaccine response titers and persistence (36).

### 2.3 Core hub: gut microbiota dysbiosis

**Figure 1:**
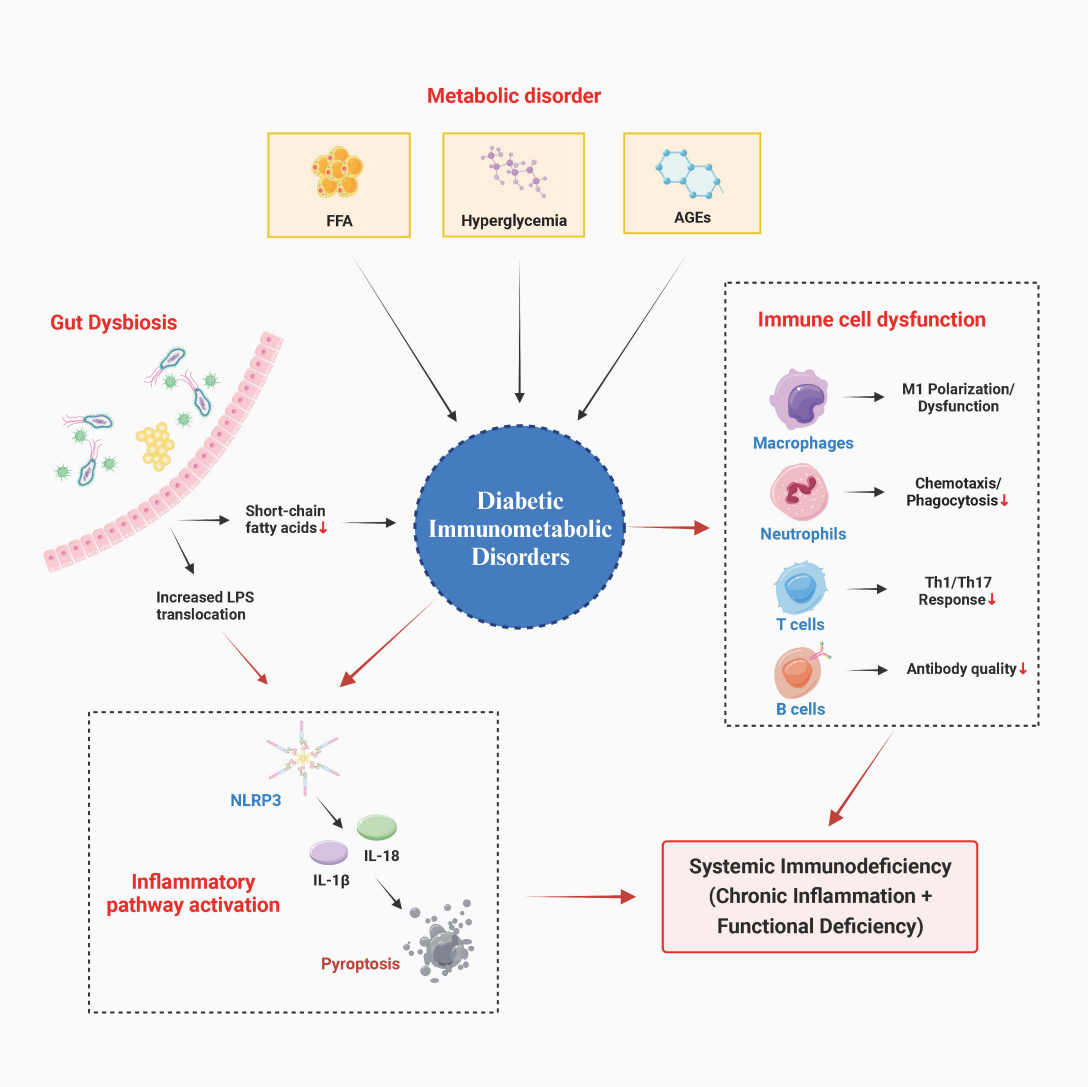
Pathophysiological network of diabetic immunometabolic disorders. This diagram illustrates how metabolic dysregulation (hyperglycemia, lipotoxicity, AGEs) in the diabetic state functions as an endogenous danger signal, directly impairing immune cell function and activating inflammatory pathways (e.g., NLRP3). Gut dysbiosis amplifies this process through the production of metabolic byproducts and the disruption of the intestinal barrier. Collectively, these factors lead to systemic immune deficiency, characterized by chronic inflammation and impaired immune responses. AGEs, Advanced glycation end products; FFA, Free fatty acids; IL-1β, Interleukin-1 beta; IL-18, Interleukin-18; LPS, Lipopolysaccharide; NLRP3, NLR family pyrin domain containing 3; Th1, T helper type 1 cells; Th17, T helper type 17 cells.

The gut microbial ecosystem plays a pivotal role in amplifying and integrating this pathological network. Diabetic patients frequently exhibit dysbiosis, characterized by a decline in microbial diversity, a decrease in the population of short-chain fatty acid-producing commensals (e.g., Prevotella, Ruminococcus), and an increase in the prevalence of opportunistic pathogens (pathogens that typically do not cause disease in immunocompetent individuals but can cause infections in immunocompromised states, such as Candida and Mucor) (37–39). This ecological imbalance interacts with host dietary and metabolic states. A primary consequence of this process is the compromise of intestinal barrier integrity, allowing the translocation of microbial-associated molecular patterns (MAMPs), such as bacterial lipopolysaccharides, into the systemic circulation. These substances have been shown to induce persistent low-grade inflammation by activating immune cells within the portal venous system and throughout the body (37–39). Concurrently, a reduction in the production of beneficial metabolites—particularly short-chain fatty acids (SCFAs) such as butyrate and propionate—weakens their regulatory effects on intestinal mucosal immunity (e.g., promoting regulatory T cell differentiation) and their activation of systemic anti-inflammatory pathways (37–39). Furthermore, dysbiosis has been shown to alter bile acid metabolic profiles, thereby influencing host glucose metabolism and inflammatory responses via nuclear receptors, such as the farnesoid X receptor (37–39). Consequently, the gut microbiota can be regarded as a pivotal “immunometabolic interface” that links exogenous dietary factors, endogenous metabolic states, and systemic immune inflammation (**Figure 1**).

In summary, the immunometabolic dysregulation in diabetes represents a multi-layered pathological process. The etiology of the condition is attributed to abnormalities in glucose, lipid, and protein metabolism. These aberrant metabolites function as signaling molecules, exerting a direct impact on immune cells, disrupting their energy metabolism and normal functions. This results in systemic dysregulation of both innate and adaptive immunity, manifesting as immune dysfunction against a backdrop of chronic inflammation. Gut microbiota dysbiosis permeates this process, amplifying and integrating the vicious cycle between metabolism and immunity. The aforementioned pathological network of immune-metabolic dysregulation does not indiscriminately weaken host defenses. Instead, it has been demonstrated to influence the susceptibility profile of diabetic patients to various pathogens through specific mechanistic pathways. These mechanistic associations reveal that diabetes does not merely compound infection risks; it fundamentally alters the onset, progression, and outcomes of infections by reshaping both the quality and quantity of the host immune response.

## 3 Infectious disease implications under the new paradigm—reassessing specific infections

Redefining diabetes as an immunometabolic disorder provides a mechanistic framework that transcends superficial observations to elucidate its complex relationship with infectious diseases. This viewpoint necessitates a shift in the prevailing paradigm, in which elevated infection risk is predominantly attributed to hyperglycemia or vascular complications. Instead, a reexamination of the profound impact of diabetes on infectious disease epidemiology, clinical phenotypes, and treatment outcomes is necessary. This examination must address the fundamental reshaping of the host’s systemic immune defense network.

### 3.1 Broad effects on susceptibility to pathogens

Firstly, diabetes-related immune-metabolic dysregulation broadens susceptibility to multiple pathogens (1). This phenomenon does not stem from a single defensive flaw but rather from a multi-layered, synergistic immune dysfunction (40). A compromised chemotaxis, phagocytosis, and killing functions of innate immune cells weaken early pathogen clearance (20). The delayed initiation and diminished efficacy of adaptive immune responses, along with impaired memory formation, prevent the establishment of effective and enduring specific defenses (30, 31). Concurrently, chronic inflammation and sustained activation of pathways such as the AGEs-RAGE axis have been observed to damage the tissue microenvironment, thereby facilitating the colonization and spread of pathogens (24). Consequently, diabetic patients exhibit heightened susceptibility not only to community-acquired and hospital-acquired infections caused by common pathogens (e.g., Staphylococcus aureus, Escherichia coli) (16, 41), but also to viruses such as influenza and respiratory syncytial virus, as well as to opportunistic fungi such as Candida and Mucor (16, 42). This wide range of susceptibility is indicative of their systemic immunodeficiency.

### 3.2 Altering the natural course and severity of infection

Within this framework, the clinical course of specific infections exhibits distinct characteristics in diabetic patients. A notable example is the novel coronavirus infection (COVID-19), which underscores the critical role of diabetes as a major risk factor for severe outcomes and mortality (43, 44). The new paradigm posits that immune dysregulation in the diabetic context is the core mechanism. On the one hand, the presence of preexisting chronic inflammatory conditions may serve as a catalyst for viral replication. Concurrently, the potential upregulation of angiotensin-converting enzyme 2 (ACE2) receptor expression may increase opportunities for viral entry, thereby collectively engendering conditions conducive to viral replication and severe disease progression (45, 46). Of particular concern is the heightened susceptibility of diabetic patients with an imbalanced immune system to uncontrolled excessive inflammatory responses (i.e., “cytokine storm”) when encountering SARS-CoV-2, which can result in acute respiratory distress syndrome and multiple organ failure (45, 46). Concurrently, hyperglycemia and endothelial dysfunction jointly exacerbate coagulation abnormalities, increasing the risk of thromboembolism (47). In the context of tuberculosis (TB), the prevalence of diabetes in conjunction with TB has emerged as a significant global concern (48–50). In diabetic patients, immunodeficiency, particularly the activation and dysfunction of macrophages and the impairment of Th1-type cellular immune responses, results in incomplete granuloma formation and reduced bactericidal capacity (51–53). This condition renders individuals more susceptible to reactivation of latent infections, accelerated progression of active TB, increased vulnerability to pulmonary cavitation, and elevated bacterial loads in the lungs, thereby amplifying their infectious potential (54–56). In the context of invasive fungal infections, the metabolic milieu, characterized by hyperglycemia, acidosis, and elevated free iron, creates a favorable environment for fungal proliferation (57, 58).

### 3.3 Influencing treatment response and outcomes of infection

**Figure 2:**
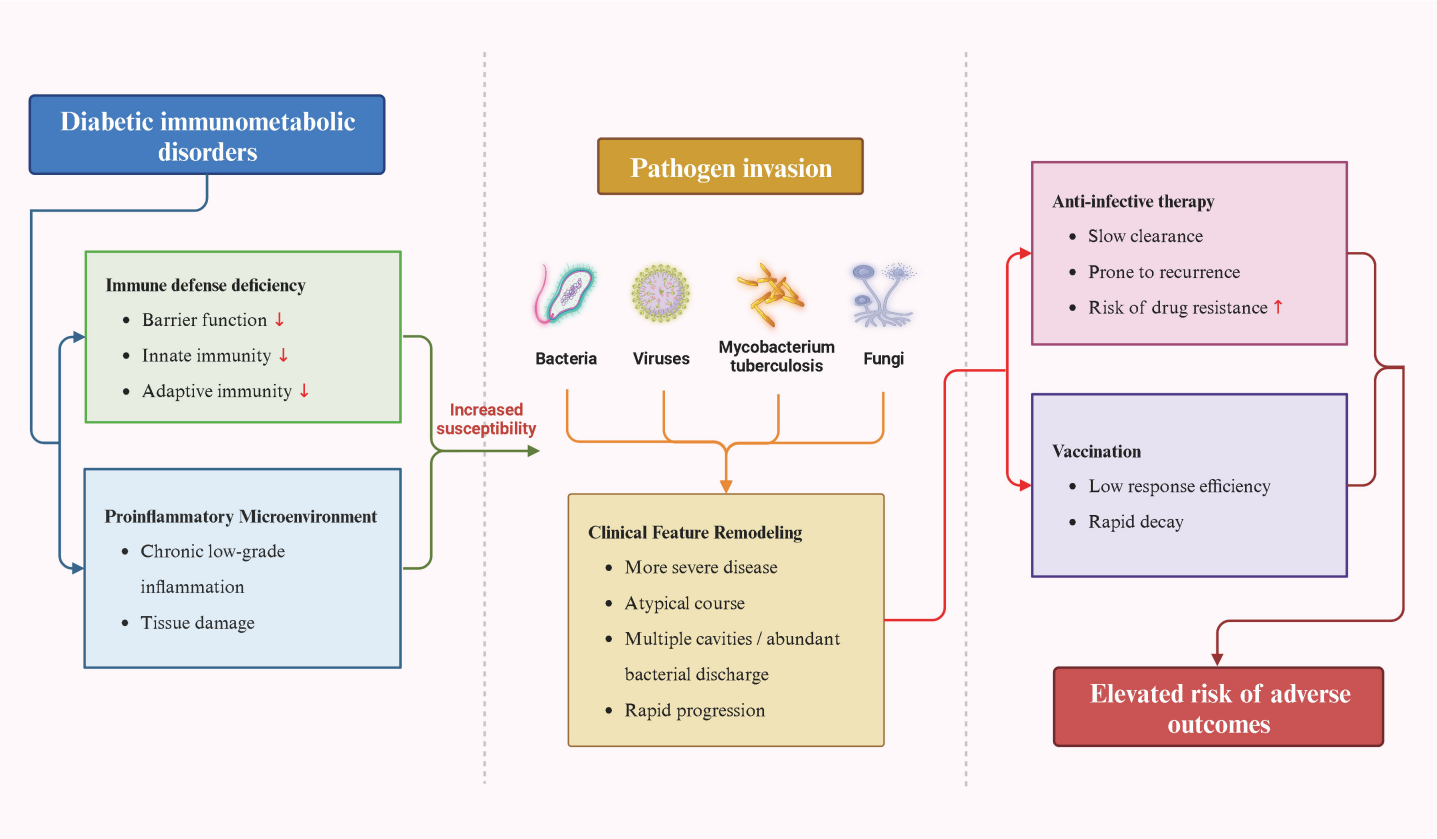
Impact of diabetes as an immunometabolic disorder on the entire clinical cycle of infectious diseases. This diagram systematically illustrates how diabetes-related systemic immune deficiencies and chronic inflammation interact to increase susceptibility to multiple pathogens and reshape the clinical course and severity of infections. Concurrently, this immunometabolic foundation also diminishes the efficacy of therapeutic and preventive interventions, ultimately leading to poorer infection outcomes.

This immunometabolic foundation profoundly influences both the response to treatment and the ultimate outcomes (2). Infection manifestations in diabetic patients may be atypical, leading to delayed diagnosis. During antimicrobial therapy, a fundamental compromise in host clearance capacity results in slower clinical responses, even with sensitive agents. This, in turn, increases susceptibility to prolonged infections, recurrence, or biofilm-associated drug-resistant infections (59). In the context of tuberculosis treatment, diabetic patients have been observed to demonstrate reduced blood concentrations of first-line drugs, such as rifampicin. When combined with impaired immune synergism, these factors can prolong the time to sputum culture conversion, increase treatment failure and recurrence risks, and potentially promote drug resistance development (54, 60). At the preventive level, diabetic conditions have been shown to diminish the efficacy of immune responses following vaccination (40). A multitude of studies have shown that diabetic patients exhibit lower seroconversion rates and peak antibody titers to seasonal influenza vaccines, pneumococcal polysaccharide vaccines, and SARS-CoV-2 vaccines compared with healthy controls. Antibody titers may also decline more rapidly, directly compromising vaccine efficacy in this population (40, 61). This phenomenon aligns with previously described mechanisms, including impaired B-cell function, insufficient T-cell help, and chronic inflammation impeding immune activation (36) (**Figure 2**).

It is noteworthy that although both type 1 and type 2 diabetes involve immune-metabolic disorders, their driving factors and manifestation patterns differ. Type 1 diabetes is characterized by the autoimmune destruction of β-cells, with immune dysfunction primarily manifesting as an imbalance dominated by autoreactive T cells (62), which may increase susceptibility to viral infections (e.g., enteroviruses). Conversely, type 2 diabetes is characterized by insulin resistance, leading to immune dysregulation that primarily manifests as chronic low-grade inflammation and impaired innate immune cell function, often concomitant with metabolic syndrome. While both of these factors ultimately result in an elevated risk of infection, it is important to note that the specific mechanisms and susceptible pathogen spectra may differ. For instance, individuals with type 2 diabetes have an increased risk of skin and soft tissue infections, as well as fungal infections. In contrast, those with type 1 diabetes may demonstrate an elevated vulnerability to specific viral infections. This distinction is of considerable significance for the development of precise prevention and treatment strategies.

In summary, when viewed through the lens of the new paradigm of immune-metabolic diseases, diabetes exerts comprehensive and profound effects on infectious diseases. It has been demonstrated that modifying the host’s immune defense ecosystem leads to a systematic worsening of infectious disease outcomes. This includes increased susceptibility, modified clinical course and severity of infection, and diminished effectiveness of therapeutic and preventive measures. This understanding elevates the relationship between diabetes and infectious diseases from a mere association to an intrinsic, pathophysiologically linked interaction. As demonstrated in **Table 1**, diabetic patients demonstrate an elevated risk of severe outcomes in infections such as SARS-CoV-2, tuberculosis, and influenza. The underlying mechanisms involve immune dysfunction, chronic inflammation, and alterations in the tissue microenvironment. These findings imply that clinical practice should develop individualized monitoring and intervention strategies tailored to different infection types.

**Table 1:** Clinical characteristics of common infections in the context of diabetes as an immunometabolic disease.

Infection type/pathogen	Key associated mechanisms of immunometabolic dysfunction	Altered clinical features/risks in individuals with diabetes	Key management implications
SARS-CoV-2 (COVID-19)	• Preexisting chronic inflammation, dysregulated cytokine storm, endothelial dysfunction, and hypercoagulability.	• Significantly increased rates of severe disease and mortality. • Higher incidence of ARDS and thromboembolic events.	• Highest priority for vaccination. • Close monitoring of inflammatory markers and coagulation status post-infection.
Mycobacterium tuberculosis	• Macrophage dysfunction, impaired Th1-cell-mediated immunity, poor granuloma formation.	• Higher reactivation of latent infection. • More frequent extrapulmonary TB. • Cavitary lung disease with high bacillary load, slower treatment response, higher relapse risk.	• Consider latent TB infection screening in high-burden areas or high-risk individuals. • Potential need for prolonged or adjusted anti-TB regimens.
Influenza Virus	• Weakened innate immune response, impaired T-cell function, reduced quality of antibody response.	• Increased risk of complications (e.g., pneumonia), hospitalization, and mortality.	• Annual influenza vaccination strongly recommended as standard care.
Skin & Soft Tissue Infections (e.g., S. aureus)	• Impaired skin barrier, reduced neutrophil chemotaxis and phagocytosis, local hyperglycemic environment.	• Markedly increased incidence (e.g., boils, cellulitis, postoperative infections). • Higher risk of progression to necrotizing fasciitis.	• Emphasize foot and skin care education. • Prompt and proper management of minor wounds.
Invasive Fungal Infections (e.g., Mucormycosis)	• Hyperglycemia, acidosis, and increased available iron promote fungal growth. • Neutrophil dysfunction.	• Rapid, aggressive progression with high mortality. • Often presents as rhino-orbital-cerebral or pulmonary disease.	• Aggressive glycemic control and acidosis correction are crucial. • Requires urgent diagnostic workup and immediate, potent antifungal therapy.

SARS-CoV-2, Severe acute respiratory syndrome coronavirus 2; COVID-19, Coronavirus disease 2019; ARDS, Acute respiratory distress syndrome; TB, Tuberculosis; S. aureus, Staphylococcus aureus.

## 4 Implications and transformations for clinical and public health practice

Redefining diabetes as an immunometabolic disorder signifies more than a mere theoretical update of its pathological essence; it necessitates pragmatic transformations in existing clinical management pathways and public health strategies. This transition is characterized by a shift in the management of infection risk for diabetic patients, moving from a traditional, reactive approach of glycemic control and anti-infective therapy to a proactive, forward-looking strategy of integrated immunometabolic homeostasis management.

### 4.1 Comprehensive assessment and therapeutic selection

At the clinical management level, treatment goals should expand beyond singular glycemic control to encompass the synergistic regulation of both metabolic and immune homeostasis. Conventional glycemic control models prioritize the reduction of HbA1c, with a predominant focus on averting microvascular and macrovascular complications. Conversely, integrated immunometabolic management builds upon this foundation by emphasizing the restoration of immune-metabolic homeostasis. This approach incorporates immune function assessment, chronic inflammation control, and infection risk prevention into routine management. Consequently, when formulating hypoglycemic regimens, it is imperative to systematically assess the potential impact of different drugs on immune function and infection risk, as well as their glucose-lowering efficacy and cardiovascular benefits (63). For instance, metformin has been demonstrated to enhance insulin sensitivity, activate the AMP-activated protein kinase (AMPK) pathway, suppress mitochondrial reactive oxygen species production, and exert immunomodulatory effects by alleviating chronic inflammation (64–66). Glucagon-like peptide-1 receptor agonists have been demonstrated to reduce body weight and enhance metabolism through both central and peripheral actions (67). These actions have the potential to indirectly mitigate obesity-associated chronic inflammatory states. Sodium-glucose cotransporter 2 (SGLT2) inhibitors have been demonstrated to yield substantial improvements in cardiovascular and renal outcomes (68). However, the hyperglycemic microenvironment they induce within the genitourinary tract may augment susceptibility to bacterial and Candida infections in these regions (69). Consequently, clinical decisions necessitate individualized risk-benefit assessments.

### 4.2 Proactive infection surveillance and prevention

Concurrently, establishing an active surveillance and prevention system for infections in diabetic patients is imperative. This encompasses integrating pneumococcal vaccination, annual influenza vaccination, and SARS-CoV-2 booster vaccination into standard preventive care (70–72), while considering the relevance of other vaccinations, such as hepatitis B (73). Moreover, screening for latent tuberculosis infection is advised for diabetic patients in regions with high tuberculosis burden or those exhibiting additional risk factors (74, 75). During post-infection treatment, clinicians must prepare for a wide range of scenarios, including but not limited to: immune clearance capacity impairment, altered antibiotic tissue penetration, and drug interactions (e.g., rifampicin accelerating the metabolism of certain oral hypoglycemic agents). This may necessitate more aggressive antimicrobial regimens, extended treatment durations, and closer multidisciplinary collaboration (**Table 2**).

**Table 2:** Immunometabolic characteristics and potential infection risks of common glucose-lowering medications.

Drug class	Representative agents	Main metabolic benefits & immunomodulatory potential	Potential increased infection risk or concerns	Key considerations for clinical decision-making
Biguanides	Metformin	• Improves insulin sensitivity. • Activates AMPK pathway, reduces oxidative stress, exerts anti-inflammatory effects.	• No clear evidence of significantly increased risk for specific infections.	• Considered as first-line foundational therapy. • Its potential immunomodulatory benefits are noteworthy.
GLP-1 Receptor Agonists	Liraglutide, Semaglutide, etc.	• Significant weight loss, improves glycemic and lipid profiles. • May indirectly reduce obesity-related chronic inflammation.	• Gastrointestinal side effects might (rarely) affect nutritional status. • No direct strong evidence for increased specific infection risk.	• Clear cardiovascular and renal benefits. • Positive impact on immunity likely mediated through overall metabolic improvement.
SGLT2 Inhibitors	Empagliflozin, Dapagliflozin, etc.	• Insulin-independent mechanism. • Proven cardiovascular and renal protective effects.	• Clearly increased risk of genitourinary infections (especially candidal vulvovaginitis/balanitis and bacterial urinary tract infections).	• Requires balancing clear cardiorenal outcome benefits against local infection risk. • Enhanced patient education and monitoring for susceptible individuals.
DPP-4 Inhibitors	Sitagliptin, Vildagliptin, etc.	• Glucose-dependent insulin secretion, low hypoglycemia risk. • Some studies suggest possible anti-inflammatory effects.	• Associated with increased risk of herpes virus infections in isolated case reports (causality not established). • Overall safety profile is favorable.	• Generally well-tolerated; infection risk signal is weak but warrants clinical awareness.
Insulin	Various formulations	• Most potent glucose-lowering agent, directly corrects glucotoxicity.	• Therapy-induced hypoglycemia may trigger stress responses. • Overuse leading to weight gain may exacerbate inflammation.	• Intensive glycemic control itself improves the immune milieu, but requires careful titration to avoid hypoglycemia and excessive weight gain.

AMPK, AMP-activated protein kinase; GLP-1, Glucagon-like peptide-1; SGLT2i, Sodium-glucose cotransporter-2 inhibitor; DPP-4, Dipeptidyl peptidase-4.

### 4.3 Emerging directions in public health and research

In the domain of public health and research, novel paradigms are precipitating strategic shifts. Public health policies must explicitly identify diabetic patients as a key vulnerable group for infectious disease control. National immunization programs, tuberculosis prevention guidelines, and emerging infectious disease contingency plans must explicitly address the unique needs and heightened risks of the diabetic population. These programs must ensure accessible and targeted prevention and medical resources. In research, there are three urgent priorities. Firstly, biomarker panels must be developed that comprehensively reflect patients’ immunometabolic status. This could include specific inflammatory cytokine profiles, immune cell function assays, or microbiome characteristics. These panels would be used to create personalized infection risk stratification. Secondly, there must be a deeper exploration of interventions targeting immune-metabolic pathways. Some examples of these pathways include IL-1β and the NLRP3 inflammasome (28). These interventions could serve as novel strategies to augment anti-infective measures or to improve diabetes outcomes. Thirdly, multi-omics technologies must be utilized. These technologies include metabolomics, immunomics, and microbiomics. They must be used to decipher the underlying mechanisms of infection outcome disparities within heterogeneous diabetic populations. This will inform precision medicine practices.

### 4.4 Immunometabolic management strategies in low-resource settings

It is imperative to recognize the challenges associated with the implementation of immunometabolic management measures, such as immune function testing and multi-omics analysis, in resource-constrained settings. Consequently, future research endeavors must prioritize the development of simplified, cost-effective operational tools. Examples include: The development of a micro-blood-based inflammatory biomarker detection method; the integration of portable blood glucose monitors with infection early-warning systems; and the leveraging of existing public health platforms (e.g., tuberculosis control networks) for combined diabetes-infection screening. At the policy level, efforts should integrate diabetes management into existing infectious disease control systems to maximize resource sharing and cost-effectiveness.

In summary, this new paradigm requires clinicians and public health policymakers to update their cognitive frameworks, integrating diabetes management into the broader context of infectious disease control. The implementation of comprehensive management strategies that integrate metabolic regulation, immune surveillance, and infection prevention, in conjunction with the promotion of corresponding scientific and policy innovations, has the potential to markedly improve the dual burden of disease faced by individuals with diabetes and enhance their health outcomes.

## 5 Challenges, controversies, and future outlook

While redefining diabetes as an immunometabolic disorder provides a robust integrative framework for understanding its interactions with infectious diseases, translating this theoretical paradigm into widespread clinical practice and public health policy faces a series of practical challenges and unresolved academic controversies.

### 5.1 Immunometabolic heterogeneity in diabetes

First, it is important to acknowledge the significant heterogeneity of diabetes, which profoundly influences its immunometabolic phenotype. Individuals with type 1 diabetes, type 2 diabetes, and varying disease duration, ages, obesity levels, and complication statuses may exhibit fundamentally different primary drivers of immune dysregulation, levels of inflammatory pathway activation, and characteristics of immune cell dysfunction (15, 76, 77). This heterogeneity makes it exceptionally challenging to identify universal immune intervention targets or single biomarkers.

### 5.2 Clinical translation challenges for biomarkers

Secondly, the prevailing clinical instruments used to evaluate patient immune function are predominantly limited to research settings, with a paucity of standardized, readily accessible, and cost-effective markers for routine diagnostic and therapeutic applications in infection risk stratification. Treatment dilemmas also exist: while intensive glycemic control may improve certain immune parameters, the impact of specific hypoglycemic agents on particular infection risks remains incompletely understood, often requiring clinical trade-offs between metabolic benefits and potential infection hazards. Moreover, the long-term safety, efficacy, and net effect on infection risk of specific therapies targeting immune-metabolic pathways (e.g., IL-1β inhibitors) in diabetic patients require validation through large-scale clinical trials (28).

### 5.3 Real-world controversies in interventions

Contemporary research endeavors are characterized by a series of contentious debates focused on several pivotal domains. Firstly, there is ongoing discourse about the relative significance and fundamental differences in immune characteristics across different diabetes subtypes. While chronic inflammation is a hallmark of type 2 diabetes, the origin and nature of immune dysregulation in type 1 diabetes—an autoimmune disorder—remain inconclusive regarding whether they confer a distinct susceptibility spectrum to infections. Secondly, there is conflicting evidence regarding the effects of specific interventions. For instance, SGLT2 inhibitors present a trade-off between potential systemic anti-inflammatory benefits and a clear increase in genitourinary infection risk, sparking debate over their net clinical value. Moreover, the efficacy of interventions targeting the gut microbiota—including probiotics, prebiotics, and fecal microbiota transplantation—in enhancing metabolic function and immune status in individuals with diabetes requires the accumulation of more robust, reproducible scientific evidence. The attainment of personalized precision regulation remains an objective for future endeavors.

### 5.4 Comparison with other chronic diseases involving immunometabolic disorders

It is important to note that diabetes is not the sole chronic disease that manifests as an immunometabolic disorder. Human immunodeficiency virus (HIV) infection is a paradigmatic comparative case, involving the interplay of immunodeficiency and metabolic abnormalities (78). The virus directly attacks CD4+ T cells, leading to acquired immunodeficiency. Antiretroviral therapy-associated metabolic syndrome (such as dyslipidemia and insulin resistance) further exacerbates the immunometabolic burden. In contrast to diabetes, the immune deficiency associated with HIV has a well-defined pathogenetic driver, and its metabolic abnormalities are more treatment-related than disease-specific. Another comparable example is rheumatoid arthritis, a chronic disease centered on autoimmune inflammation. In this case, metabolic abnormalities (such as insulin resistance and dyslipidemia) primarily result from systemic inflammation and glucocorticoid therapy (79). Conversely, the immunometabolic disruption observed in type 2 diabetes manifests a “primary” characteristic, wherein metabolic abnormalities serve as the driving force, while immune inflammation is the outcome. It is noteworthy that both factors are mutually causative. This distinction dictates differing intervention strategies: diabetes management should prioritize metabolic regulation to enhance immune function, whereas HIV and rheumatoid arthritis require controlling the primary disease while managing secondary metabolic issues.

### 5.5 Future directions: precision medicine and multidisciplinary integration

In the future, research and practice in this field will evolve toward greater precision, integration, and interdisciplinarity. The field of precision medicine is evolving to include applications in the management of diabetes and its immunometabolic complications. For instance, there is ongoing exploration of infection risk stratification based on patient immunometabolic phenotypes. Researchers employ a multifaceted approach to patient classification, encompassing the categorization of patients into subtypes such as “high-inflammation” or “immune-exhausted” through the analysis of cytokine profiles (e.g., IL-1β, TNF-α levels), immune cell function (e.g., neutrophil phagocytic activity), and gut microbiota characteristics. An additional illustration of this phenomenon is the utilization of a customized selection process for SGLT2 inhibitors. For patients with recurrent urinary tract infections, clinicians can weigh cardiovascular benefits against infection risks based on infection history, urine culture results, and microbial profiles to choose alternative medications or enhance preventive measures. These cases illustrate the promise of precision interventions, guided by immune-metabolic characteristics, to augment therapeutic efficacy and mitigate infection risks. At the drug development level, a key trend will be the optimization of immunological safety assessments for existing hypoglycemic agents. Concurrently, novel therapies will be developed that enhance insulin sensitivity, reduce chronic inflammation, and restore immune homeostasis. Technological integration of multi-omics data (including metabolomics, proteomics, single-cell immunomics, and metagenomics) will profoundly deepen our understanding of diabetes’s complex immunometabolic networks and facilitate the discovery of novel biomarkers and therapeutic targets. Finally, the advancement of interdisciplinary collaboration is of the utmost importance. Future efforts must entail more robust collaboration mechanisms among experts in endocrinology, infectious diseases, immunology, microbiology, and public health. A collaborative approach involving the design of clinical trials, the formulation of comprehensive management guidelines, and the optimization of healthcare systems will facilitate a systematic response to the dual challenges posed by diabetes and infectious disease comorbidity. In essence, integrating immune-metabolic health as a fundamental objective in diabetes management has the potential to effect a pivotal shift from the prevailing paradigm of “controlling blood glucose to prevent complications” to a new model of “restoring immune-metabolic homeostasis to enhance overall health and defense capabilities (**Table 3**).”

**Table 3:** Key challenges and future directions in the field of diabetes immunometabolism and infection research.

Core area	Current major challenges & controversies	Future research directions & potential strategies
Heterogeneity & Precise Phenotyping	• Vast immunometabolic heterogeneity between T1D, T2D, and individuals. • Lack of universal biomarkers.	• Utilizing multi-omics (single-cell sequencing, metabolomics, metagenomics) for endotype classification. • Developing infection risk prediction models based on immunometabolic profiles.
Clinical Translation & Assessment Tools	• Lack of standardized, clinically feasible immune function assays for guiding individualized management.	• Developing and validating simplified immune assessment panels (e.g., specific cytokine profiles, functional immune cell assays). • Exploring feasibility of wearable devices for monitoring inflammatory markers.
Precision in Pharmacological Intervention	• Balancing systemic metabolic benefits of some glucose-lowering drugs against localized/specific infection risks (e.g., SGLT2i). • Unclear long-term benefit-risk profile of immunomodulatory therapies (e.g., anti-IL-1β).	• Conducting large-scale, prospective studies to define the “net infection risk” of various drug classes. • Designing trials to assess efficacy of immune-targeted therapies in preventing or adjunctively treating infections in diabetes.
Interdisciplinary Integration in Practice	• Relative siloing of endocrinology, infectious diseases, and immunology in both clinical care and research.	• Establishing multidisciplinary clinics and research consortia; developing integrated clinical practice guidelines merging metabolic, immune, and infection control principles. • Designing public health-level integrated prevention programs.

T1D, Type 1 diabetes; T2D, Type 2 diabetes; SGLT2i, Sodium-glucose cotransporter-2 inhibitor; IL-1β, Interleukin-1 beta.

## 6 Conclusion

This review methodically substantiates the theoretical value of redefining diabetes as an “immunometabolic disorder.” This framework challenges traditional perspectives by addressing the fundamental mechanisms of immune-metabolic cross-regulation, thereby providing a unified pathophysiological explanation for the high susceptibility to infection, severe disease progression, and poor treatment outcomes observed in diabetic patients. The innovative contributions of this work manifest on three levels. Firstly, it constructs an integrated explanatory framework for diabetes-infection interactions. Secondly, it elucidates how metabolic dysregulation—including hyperglycemia and lipotoxicity—leads to immune dysfunction through shared pathways. Thirdly, it proposes a clinical paradigm shift from “glycemic control” to “immune-metabolic homeostasis management.” In light of these findings, we urge researchers to prioritize the study of immune-metabolic phenotypes and the development of precision interventions. We also advocate for clinicians to incorporate immune assessment into their standard management practices. Furthermore, we recommend that policymakers consider diabetic patients as a priority group in the context of infectious disease prevention and control measures. The most effective approach to addressing the dual challenges posed by diabetes and infectious disease comorbidity is through multidisciplinary collaboration.
